# ANGSD: Analysis of Next Generation Sequencing Data

**DOI:** 10.1186/s12859-014-0356-4

**Published:** 2014-11-25

**Authors:** Thorfinn Sand Korneliussen, Anders Albrechtsen, Rasmus Nielsen

**Affiliations:** Centre for GeoGenetics, Natural History Museum of Denmark, Copenhagen, Denmark; Bioinformatics Centre, Department of Biology, University of Copenhagen, Ole Maaloes Vej 5, Copenhagen, DK-2200 Denmark; Department of Integrative Biology and Statistics, UC-Berkeley, 4098 VLSB, Berkeley, California, 94720 USA

**Keywords:** Next-generation sequencing, Bioinformatics, Population genetics, Association studies

## Abstract

**Background:**

High-throughput DNA sequencing technologies are generating vast amounts of data. Fast, flexible and memory efficient implementations are needed in order to facilitate analyses of thousands of samples simultaneously.

**Results:**

We present a multithreaded program suite called ANGSD. This program can calculate various summary statistics, and perform association mapping and population genetic analyses utilizing the full information in next generation sequencing data by working directly on the raw sequencing data or by using genotype likelihoods.

**Conclusions:**

The open source c/c++ program ANGSD is available at http://www.popgen.dk/angsd. The program is tested and validated on GNU/Linux systems. The program facilitates multiple input formats including BAM and imputed beagle genotype probability files. The program allow the user to choose between combinations of existing methods and can perform analysis that is not implemented elsewhere.

**Electronic supplementary material:**

The online version of this article (doi:10.1186/s12859-014-0356-4) contains supplementary material, which is available to authorized users.

## Background

Next generation sequencing (NGS) platforms can generate large amounts of sequencing data, but often with high sequence error rates. For low to medium depth data fast and efficient implementation are needed to handle the data. Arguably, downstream analyses should be performed in a probabilistic context by working with the raw data in form of genotype likelihoods (GL) [1]. ANGSD is a novel and efficient program that allows for multiple error models used within the GL calculation. The remainder of this section describe the typical work flow used for analyzing data. The implementation section lists and describes existing (published) methods and new methods that are available in our tool. The majority of methods in ANGSD are not implemented in other software and in the results section we have therefore limited the comparisons with existing tools to basic analyses of SNP-discovery and genotype calling.

The first step in a bioinformatic pipeline for analyzing NGS data is usually to align the reads to a reference genome using a fast short read aligner [2-5]. State-of-the-art alignment programs will, in addition to inferring the genomic start position of the reads, provide additional information such as the mapping quality scores (mapQ), and possibly also indicate which parts of an alignment may be affected by indels. Information regarding sequencing quality is included in quality scores (qscores), typically provided by the sequencing technology, and often modified using downstream re-calibration [6-8]. Based on the aligned reads, and associated mapping and sequencing quality scores, a genotype likelihood (GL) is then calculated. The GL is (up to a scaling factor) the marginal probability of the sequencing data given a genotype in a particular individual, in a particular site. Most data analyses then proceed by calling SNPs and genotypes from the GLs, typically combining information from multiple individuals, often also combining the GL with prior information, such as the inferred distribution of allele frequencies. For many applications based on high-quality deeply sequenced data, this is a near-optimal strategy for analyzing the data. However, for low or medium coverage data, there is often a distinct statistical advantage in working on the raw data, or GLs, rather than called genotypes in downstream analyses [9-14]. Working directly on GLs facilitates the incorporation of statistical uncertainty regarding genotypes. The uncertainty regarding genotypes in low coverage data arises from several sources, including mapping and sequencing errors, and the random sampling of (haploid) reads from a diploid genotype.

The *de facto* standard format to store and distribute NGS data in, is the BAM format which allows for random access within the sequencing data. When analyzing many individuals simultaneously, due to memory constraints, it is often convenient to analyze regions or single sites independently instead of reading all the data into memory. This is achieved by reading parts of each BAM file, aligning and then passing the aligned sites for analysis. Here we present an open source mutithreaded C/C++ program called ANGSD with this capability. ANGSD provides easy user access to methods for population genetic analyses and association mapping utilizing the full information of the data and taking uncertainty regarding SNP calling and genotype calling into account, by working directly on user-provided, or *de novo* estimated, GLs.

Examples of existing general multisample NGS analysis programs are the singlethreaded SAMtools [15] (C) and the multithreaded GATK [8] (Java). There are many differences between the three programs, but the key advantage with ANGSD is that it 1) allows for multiple input data types relating directly to raw sequencing data (text mpileup, binary genotype likelihood files, VCF files), 2) allows the user to choose between multiple methods for intermediate analysis such as different ways to calculate GL and 3) includes implementations of a large set of downstream analyses not implemented in any other software.

## Implementation

### Input formats

**Figure 1:**
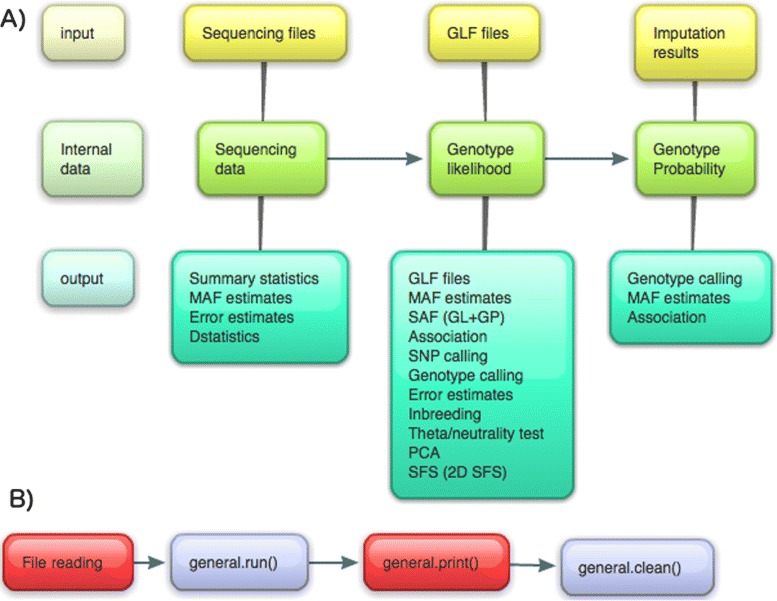
**Data formats and call graph.** **A)** Dependency of different data formats and analyses that can be performed in ANGSD. **B)** Simplified call graph. Red nodes indicate areas that are not threaded. With the exception of file readers, all analyses, printing and cleaning is done by objects derived from the abstract base class called general.

ANGSD can currently parse a variety of different input formats including binary BAM files and mpileup text files. Genotype likelihoods input are supported for simple genotype likelihood formats and it also supports genotype (posterior) probabilities in the BEAGLE [16] format. ANGSD can perform various analyses, but some of these can be limited by the chosen input format e.g., sequencing depth calculation can only be performed on the basis of raw sequencing files and not GLs. The dependency between the different analysis and input formats is depicted in Figure 1. Indexed BAM files facilitate random access and this feature is implemented in ANGSD. Random access is not supported for other file formats.

### Methods

Some analyses can be performed on a single-site basis such as simple allele frequency estimation (MAF) and site-wise association testing. For analyses on genome wide data, the work-flow is divided into two steps: 1) ANGSD generates specific input data for the analysis. 2) A secondary associated program is used to perform the downstream analysis based on the ANGSD output [17]. For simple tests such as ABBA-BABA/D-statistic [18] the secondary program can be a simple Rscript, but for computational intensive methods it can be a multithreaded c/c++ program. A description of the main methods are found in Table 1. ANGSD allows a number of different types of input data, but not all analyses can be performed if the input data is not sequencing data. For example, only a few methods are applicable if the input data is genotype probabilities, e.g., likelihood ratio test for SNP calling can only be performed on GLs and not genotype probabilities (posteriors probabilities) (see Figure 1).

**Table 1:** **Overview of analyses implemented in ANGSD**

**Analysis**	**Basis**	**Reference**
**Contamination estimates** based on the X-chromosomes	BC	[19] ^*b*^
**Type specific error estimation** estimated by simultaneously estimating allele frequencies and genotype likelihoods	GL	[10]
**Type specific error estimation** based on an outgroup and a high quality genome	BC	[20] ^*ab*^
**Genotype likelihoods (GL)** (diploids)	BC/Seq	[6,8,10,15]
**Allele frequencies** for a site	BC/GL/GP	[21] ^*b*^ [10]
**SNP discovery (LRT)** used for rejecting that the allele frequency is different from zero	GL	[10]
**Genotype posteriors (GP)** can be used for calling genotypes by specifying a cutoff	GL/SAF	[9,10]
**Sample allele frequencies (SAF)** the probability of all read data given the sample allele frequency	GL/GP	[9] ^*b*^
Population differentiation statistics ***F***_***st***_	SAF	[14] ^*ac*^
Population structure via principle components analysis **(PCA)**	GP	[14] ^*ac*^
**Admixture analysis (NGSadmix)** NGS data	GL	[22] ^*ab*^
Detection of ancient admixture **ABBA-BABA/d-statistics**	BC	[20] ^*b*^
Estimation of **SFS (1D)**	SAF	[9] ^*ab*^
Estimation of **SFS (2D)**	SAF	
**Selection scans**, Neutrality tests (e.g *θ*’s and Tajima’s D)	SAF	[12] ^*ab*^
Estimation of individual and site-wise **Inbreeding** coefficients. Also MAF and GP estimation for inbreed individuals	GL	[13] ^*abc*^
**Allele frequency based association** for case/control data)	GL	[10]
**Association score test** in a generalized linear model framework for both quantitative and case/control data while allowing for additional covariates	GL-GP	[11] ^*b*^

Table of the supported analyses in ANGSD. ^*a*^indicates methods that require a secondary program in ANGSD package. ^*b*^indicates methods for which ANGSD is the *de facto* implementation and ^*c*^are user supplied extensions for ANGSD. The basis for each analysis is either the sequencing data (Seq), base counts (BC), genotype likelihood (GL), sample allele frequencies (SAF) or genotype probabilities (GP).

#### Genotype likelihoods

For low and medium coverage NGS data, the recommended practice is to avoid basing downstream analysis on the raw counts of sequenced bases or called genotypes [1], but instead use a probabilistic approach by using GLs. Many of the methods within ANGSD are based on GLs (Table 1). ANGSD supports four different models for calculating GLs: 1) The recalibrating SOAPsnp model [6]. 2) The original GATK model [8] 3) SAMtools 1.16+ modified Maq model [23]. 4) The type specific error model [10]. The sequencing error rates in these GL models are either fixed, obtained from qscores, or estimated from the data. The four implemented GL models assume diploid samples.

#### Allele frequency estimators

The sample allele frequency in a site is the frequency of the allele among the individuals included in a specific sample. The population allele frequency is the (unknown) frequency of the allele in the entire population. Without genotype uncertainty, the sample allele frequency is known and the population allele frequency can be estimated from the sample allele frequency. However, in the presence of genotype uncertainty, the sample allele frequency is unknown, but can be estimated from the raw data or from the genotype likelihoods [9]. We have implemented several estimators of population (e.g., [10,21]) and sample allele frequencies (e.g., [9]), that can be estimated based on GL’s, base counts or genotype posteriors. By using the population allele frequency we have implemented a *likelihood ratio test* (LRT) of the site being variable which can be used as a SNP discovery criterion, and a Bayesian approach for calling genotypes.

#### Population genetic analysis based on sample allele frequencies

Several analyses are based on sample allele frequency likelihoods instead of single individual genotype likelihoods. A sample allele frequency likelihood is (up to a scaling factor) the probability of all read data for multiple individuals at a site, given the sample allele frequency. The methods in [9] use the sample allele frequency likelihood in several applications, including estimation of the site frequency spectrum (SFS), and estimation of Tajima’s D and various other neutrality tests can be estimated taking genotype uncertainty into account [12]. These methods are included in the ANGSD package as separate programs that utilize ANGSD output. Various Bayesian estimation procedures are also implemented, including maximum *a posteriori* probability (MAP) estimates of the sample allele frequency [9]. The implementation in ANGSD allows for the use of externally estimated posterior probabilities (obtained for example using haplotype imputation based methods) for the calculation of posterior sample allele frequencies and other downstream analyses. Importantly, ANGSD also allows for the joint estimation of sample allele frequencies from two populations (2D-SFS):

Assuming two populations with *n*_1_ and *n*_2_ diploid individuals sampled from population 1 and 2, respectively. Then the 2D-SFS is the matrix *γ* :(2*n*_1_+1)×(2*n*_2_+1) of frequencies of derived sample allele counts in the two populations, i.e. *γ*_*ij*_ is the probability of observing *i* and *j* derived alleles population 1 and 2, respectively, in a randomly chosen site.

Let $p\left ({X^{d}_{s}}\mid D_{d}=i\right)$ denote the likelihood for the sequencing data, in population d for site *s*, given a total of *i* derived alleles in population *d*. This likelihood is calculated using the algorithm described in ref. [9]. We can then write the likelihood for a single site *s* for the 2D-SFS as:

(1) $${} \begin{aligned} L\left(X|\gamma\right)=\prod\limits_{s=0}^{N}L\left(X_{s}\mid \gamma\right) &= \prod\limits_{s=0}^{N} \sum\limits_{i=0}^{2n_{1}}\sum\limits_{j=0}^{2n_{2}} \gamma_{ij}p\left({X^{1}_{s}}\mid D^{1}=i\right)\\& \quad \times p\left({X^{2}_{s}}\mid D^{2}=j\right) \end{aligned}  $$

In order to find the maximum likelihood we use an EM-algorithm. Assuming *γ*^*old*^ is our current parameters, a next iteration in the EM-algorithm is given by:

$${} \gamma_{ij}^{new} = \sum\limits_{s=0}^{N} p\left({X_{s}^{1}}\mid D^{1}=i\right)p\left({X_{s}^{2}}\mid D^{2}=j\right) / L\left(X_{s}\mid \gamma^{old}\right). $$

The algorithm then iterates updates of all *ij* simultaneously until the difference in successive likelihood values is below some tolerance.

#### Population structure

Genomes for admixed individuals represent a mixture of alleles from different ancestral populations. Inferring individual admixture proportions along with a frequency estimate for the different ancestral populations is possible based on genotype likelihoods [22] based on output from ANGSD. Similarly the sample allele frequency likelihoods generated in ANGSD can be used to calculate statistics relevant to population structure analyses including inbreeding coefficients [13], *F*_*st*_ and principal component analyses (PCA) [14].

Another approach for detecting admixture including ancient admixture is the ABBA-BABA test also called the D-statistic [18]. For sequencing data the strategy for this test is based on sampling a single base at each position of the genome [24]. This strategy removes bias caused by depth differences which is a fundamental problem of NGS data. Given an outgroup ANGSD gives D-statistics for all possible combinations of the chosen individuals.

#### Association

Finding disease causing mutation is often done using association studies based on called genotypes. ANGSD provides two approaches for performing association studies that are appropriate for NGS data. Both are based directly on genotype likelihoods which takes all the uncertainty of the NGS data into account. The first method can be used in a simple case/control setting [10] where differences in allele frequencies between cases and controls are compared. The other approach is a more flexible generalized linear regression framework [11] which allows for quantitative traits and inclusion of covariates. This approach is also implemented for genotype probabilities such as the ones estimated from haplotype imputation.

#### Base error estimation

Several error estimates of type specific base error rates are implemented. The simplest is based on the mismatch rate that also forms the basis of SOAPsnp [6]. Another approach that tries to estimate the real error rate and not the mismatch rate is based on an outgroup [20,24] and a high quality individual. The third approach estimates error rates, genotype likelihoods and allele frequencies simultaneously in order to determine the base error rate of polymorphic sites [10].

#### Limitations & roadmap

Most statistical methods in ANGSD assume a diploid organism and does not support pooled data. Indels are represented internally in ANGSD, but no method currently utilizes this information. We also acknowledge that bcf/vcf files are heavily used and have begun including basic vcf input/output in the development version. No analysis in ANGSD uses pedigree information such as GATK’s **PhaseByTransmission**. The CRAM format has been suggested as a successor to the BAM format, but ANGSD does not support this in the current version and depending on the general acceptance of this new format we might include it in future versions. Finally SAMtools and GATK include many different filters at the site level whereas these have not been included in ANGSD yet.

## Results and discussion

ANGSD is the *de facto*(sole) implementation of many published methods (see Table 1), and we will in this section show examples of how to use ANGSD including a novel method of estimating the joint site frequency spectra for two populations and an implementation of the ABBA-BABA D-statistic [18] for NGS data. We will also show that having the ability to decide which method to use for some of the intermediate analyses, such as calculation of GL, is important and can have a large effect on the downstream analyses.

### The genotype likelihood model affects downstream analysis

As an example of the effect of genotype likelihood model on the analysis, we estimated the SFS for 12 European (CEU) and 14 African (YRI) unrelated samples from the 1000 genomes project [25] sequenced using the Illumina platform. We used the method described in [9] implemented in ANGSD to estimate the site-frequency spectrum. This is a two step procedure that first involves calculating the sample allele frequency likelihoods followed by a numerical optimization for finding the maximum likelihood estimate of the SFS. Ancestral sites were obtained from the PanTro2 genome from the multiz46way dataset sync://hgdownload.cse.ucsc.edu/goldenPath/hg19/multiz46way/maf (available from the UCSC browser), and the analysis was based on a 170 Mb region from chromosome 1 by limiting our analysis to the sites with high mappability and discarding telomeres and centromeres. The ANGSD command used was



We use the BAM files for the European samples listed in the file “CEU.list”, limit our analysis to the non-centromeric/telomeric regions defined in the file “regions.txt”, estimate the sample allele frequencies likelihoods (-doSaf), define the output files (-out) with prefix ceu.gl1, and use the genotype likelihood model from SAMtools (-GL 1). In order to estimate the joint allele frequency with YRI at a later stage we restrict the printed output to sites that are also present in the African sample and specified in the filters.txt file.

We also repeat the above analysis using the YRI population, and repeat the analyses for both populations using the GATK genotype likelihood model [8] (-GL 2).

**Figure 2:**
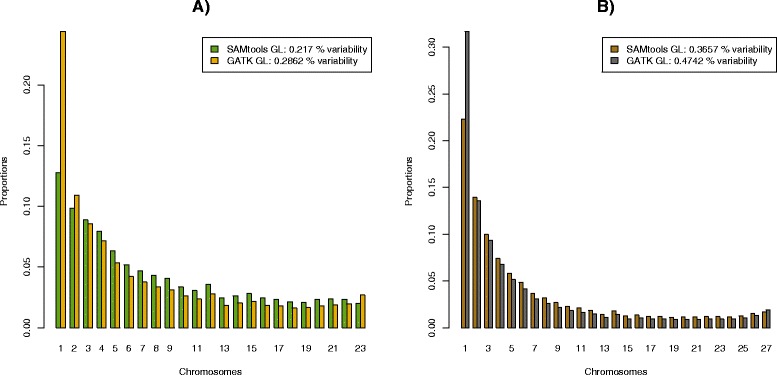
**1D SFS for different GL models.** SFS estimation based on a 170 megabase region from chromosome 1 using 12 CEU samples **A)** and 14 YRI samples **B)**” from the 1000 genomes project. The analysis was performed for both the GATK GL model (green, light brown) and SAMtools GL (yellow,dark brown). Notice the difference in estimated variability (proportion of variable sites) for the two GL models, with GATK GL based analyses inferring more variable sites and an associated larger proportion of low-frequency alleles. The two categories of invariable sites have been removed and the distributions have been normalized so that the frequencies of all categories sum to one for each method.

From the sample allele frequency likelihoods for each site we then estimate the SFS using the Expectation Maximization (EM) algorithm:



Here realSFS is the secondary program written in c++, and finds the optima of equation (5) in [9]. We supply the realSFS with the file containing the sample allele frequency likelihoods (*ceu.gl1.saf*) and tell the program that the file contains 24 chromosomes (12 diploid individuals) and it should try to use 20 computer cores. The resulting four frequency spectra (SAMtools/GATK,CEU/YRI) are shown in Figure 2. From the figure it is evident that the analysis is highly sensitive to the chosen GL model. We emphasize that there is a clear need for more research on comparing methods for estimating GLs, and possible for developing new and more appropriate methods for estimating GLs. However, such research is beyond the scope of this paper. We here emphasize that the ANGSD approach for estimating the SFS has been shown by others [26] to be superior to the genotype calling approaches used by SAMtools and GATK.

### Joint site frequency spectrum

**Figure 3:**
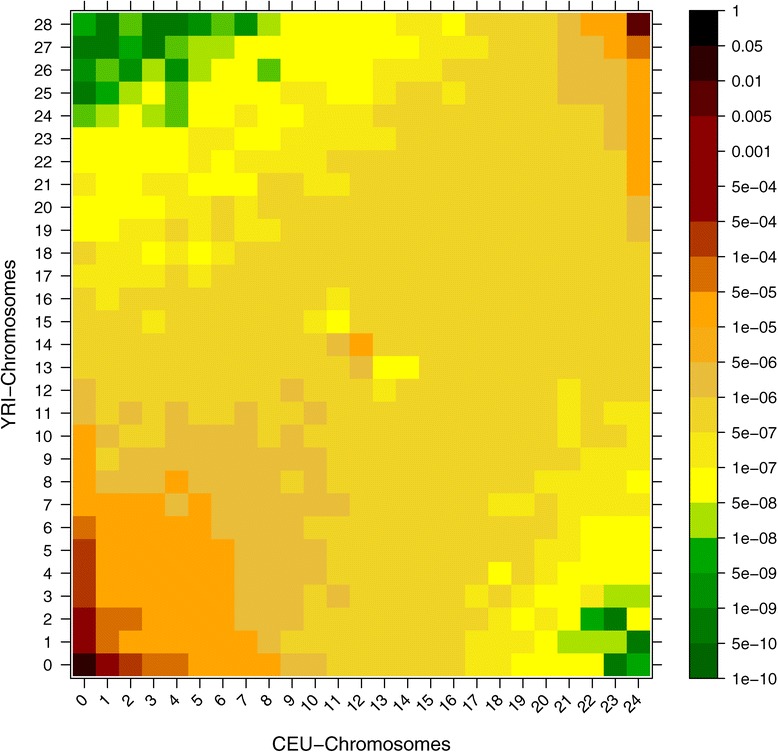
**Joint SFS (2D-SFS).** Two dimensional SFS estimation based on a 170 megabase region from chromosome 1 using 12 CEU samples and 14 YRI samples from the 1000 genomes project.

We have generalized the approach for estimating the one dimensional SFS [9] to allow for two populations (see Methods section). To obtain the maximum likelihood estimate of the joint frequency spectrum we use an EM algorithm (equation 1) by evoking the following command:



The result is shown on Figure 3. Unlike joint SFS based on SNP chip data (e.g. [27]), where most SNPs are polymorphic in both African and Europeans, this plot shows that most derived alleles are private to one of the populations. This is also observed between Chinese and Africans [28] and the difference between the SNP chip data and the sequencing data is caused by ascertainment biases in the chip data where SNPs are often chosen because they are common in populations such as European [27]. We have also performed a proper simulation study by simulating genotypes for two populations that follows a demographic pattern similar to European and African populations, assuming realistic recombination and mutation rates for humans. We simulated genotypes corresponding to a 10 Mb region using MSMS [29], and based on the genotypes we calculated genotype likelihoods using the method described in [12]. This was done by assuming a mean sequencing depth of 2X and an error rate of 0.2%. The true spectrum is visualized as a heat map in Additional file 1: Figure S1, and our estimated spectrum in Additional file 2: Figure S2 and Additional file 3: Figure S3.

### ABBA-BABA

To illustrate the use of the ABBA-BABA analyses [18,24] we demonstrate two analyses: (1) an analysis of modern human samples, and (2) a comparison of modern human sequences and ancient DNA from the Denisovan hominin. For the modern individuals we tested a European (French), a Native American (Karitiana), a Papuan (Papuan1), a Han Chinese and an African (Yoruba) [30]. The ANGSD command used in the first analysis was



The ABBA-BABA test is based on a sample of counts of bases (-doCount 1), an outgroup (-anc), which in this case is the chimpanzee, 5 Mb block size (-blockSize), and a strict filtering of bases based on quality scores (-minQ 30) and mapping quality (-minMapQ 30). A small Rscript is used to perform a blocked (uneven m-delete) jack knife procedure to obtain standard deviations and resulting the Z-scores. The results are shown in Table 2. The results are consistent with the current understanding of human migration out of Africa and even shows the recently proven link between Europeans and Native Americans through a shared ancestral central Asian population [31].

**Table 2:** **D-stat results for modern samples**

	**H1**	**H2**	**H3**	**nABBA**	**nBABA**	**Dstat**	**jackEst**	**SE**	**Z**
1	HGDP00521 (French)	HGDP00998 (American)	HGDP00927 (Yoruba)	355539	360029	-0.01	-0.01	0.00	-1.40
2	HGDP00521 (French)	HGDP00778 (Han china)	HGDP00927 (Yoruba)	361594	369006	-0.01	-0.01	0.00	-2.40
3	HGDP00998 (American)	HGDP00778 (Han china)	HGDP00927 (Yoruba)	332227	334990	-0.00	-0.00	0.00	-0.90
4	HGDP00521 (French)	HGDP00542 (Papuan1)	HGDP00927 (Yoruba)	360153	383994	-0.03	-0.03	0.00	-6.80
5	HGDP00998 (American)	HGDP00542 (Papuan1)	HGDP00927 (Yoruba)	347593	366979	-0.03	-0.03	0.00	-5.80
6	HGDP00778 (Han china)	HGDP00542 (Papuan1)	HGDP00927 (Yoruba)	347017	363467	-0.02	-0.02	0.00	-5.20
7	HGDP00927 (Yoruba)	HGDP00998 (American)	HGDP00521 (French)	653515	360029	0.29	0.29	0.00	60.60
8	HGDP00927 (Yoruba)	HGDP00778 (Han china)	HGDP00521 (French)	639280	369006	0.27	0.27	0.01	53.00
9	HGDP00998 (American)	HGDP00778 (Han china)	HGDP00521 (French)	384915	407967	-0.03	-0.03	0.01	-5.40
10	HGDP00927 (Yoruba)	HGDP00542 (Papuan1)	HGDP00521 (French)	626366	383994	0.24	0.24	0.01	43.10
11	HGDP00998 (American)	HGDP00542 (Papuan1)	HGDP00521 (French)	399343	450303	-0.06	-0.06	0.01	-10.10
12	HGDP00778 (Han china)	HGDP00542 (Papuan1)	HGDP00521 (French)	405942	433790	-0.03	-0.03	0.01	-5.50
13	HGDP00927 (Yoruba)	HGDP00521 (French)	HGDP00998 (American)	653515	355539	0.30	0.30	0.00	61.20
14	HGDP00927 (Yoruba)	HGDP00778 (Han china)	HGDP00998 (American)	711281	334990	0.36	0.36	0.01	71.80
15	HGDP00521 (French)	HGDP00778 (Han china)	HGDP00998 (American)	486385	407967	0.09	0.09	0.01	15.10
16	HGDP00927 (Yoruba)	HGDP00542 (Papuan1)	HGDP00998 (American)	660154	366979	0.29	0.29	0.01	53.80
17	HGDP00521 (French)	HGDP00542 (Papuan1)	HGDP00998 (American)	445929	450303	-0.00	-0.00	0.01	-0.80
18	HGDP00778 (Han china)	HGDP00542 (Papuan1)	HGDP00998 (American)	394958	477720	-0.09	-0.09	0.01	-15.30
19	HGDP00927 (Yoruba)	HGDP00521 (French)	HGDP00778 (Han china)	639280	361594	0.28	0.28	0.00	57.00
20	HGDP00927 (Yoruba)	HGDP00998 (American)	HGDP00778 (Han china)	711281	332227	0.36	0.36	0.01	72.70
21	HGDP00521 (French)	HGDP00998 (American)	HGDP00778 (Han china)	486385	384915	0.12	0.12	0.01	20.80
22	HGDP00927 (Yoruba)	HGDP00542 (Papuan1)	HGDP00778 (Han china)	666222	363467	0.29	0.29	0.01	55.10
23	HGDP00521 (French)	HGDP00542 (Papuan1)	HGDP00778 (Han china)	459135	433790	0.03	0.03	0.01	4.70
24	HGDP00998 (American)	HGDP00542 (Papuan1)	HGDP00778 (Han china)	401357	477720	-0.09	-0.09	0.01	-14.20
25	HGDP00927 (Yoruba)	HGDP00521 (French)	HGDP00542 (Papuan1)	626366	360153	0.27	0.27	0.01	54.00
26	HGDP00927 (Yoruba)	HGDP00998 (American)	HGDP00542 (Papuan1)	660154	347593	0.31	0.31	0.01	60.60
27	HGDP00521 (French)	HGDP00998 (American)	HGDP00542 (Papuan1)	445929	399343	0.06	0.06	0.01	9.50
28	HGDP00927 (Yoruba)	HGDP00778 (Han china)	HGDP00542 (Papuan1)	666222	347017	0.32	0.32	0.01	61.90
29	HGDP00521 (French)	HGDP00778 (Han china)	HGDP00542 (Papuan1)	459135	405942	0.06	0.06	0.01	10.40
30	HGDP00998 (American)	HGDP00778 (Han china)	HGDP00542 (Papuan1)	401357	394958	0.01	0.01	0.01	1.30

Results of the ABBABABA analysis for modern individuals from the human genetic diversity panel.

In the second analysis we used the following commands:



In the command line above, we removed transitions (-rmTrans) which are known to have extremely high error rates for ancient genomes. A more elaborate scheme for filtering bases using base quality scores can also be used to specify a different threshold for each individual and each of the four bases, and has also been implemented [24,32]. The results for the tests are shown in Table 3. This test for introgression between Papuan ancestors and Denisovans rejects the tree (((Yoruban,Papuan),Denisova), chimpanzee), with a Z score of 12.1, in accordance with the current understanding in the field [30,32].

**Table 3:** **D-stat for ancient sample**

	**H1**	**H2**	**H3**	**nABBA**	**nBABA**	**Dstat**	**jackEst**	**SE**	**Z**
1	HGDP00927 (Yoruba)	HGDP00542 (Papuan1)	T_hg19_1000g (Denisova)	103016	90667	0.06	0.06	0.01	12.10
2	T_hg19_1000g (Denisova)	HGDP00542 (Papuan1)	HGDP00927 (Yoruba)	286551	90667	0.52	0.52	0.00	127.10
3	T_hg19_1000g (Denisova)	HGDP00927 (Yoruba)	HGDP00542 (Papuan1)	286551	103016	0.47	0.47	0.01	88.60

Results of the ABBABABA analysis for 2 modern individuals and one ancient sample.

### SNP discovery and genotype calling

Population genetic analyses are traditionally based on called genotypes, but this poses a significant problem for NGS data due to the nature of the technology. Genotypes are not directly observable, but must be inferred from the data. For low or medium coverage data there can be considerable uncertainty in genotype inferences, potentially leading to errors or biases in downstream analyses. Arguably, the optimal solution to this problem is to avoid genotype calling altogether, and instead base inferences on methods that incorporate genotype uncertainty with the GLs [9-14,22,26,33]. However, we recognize that many analyses have not been generalized to be based on GLs instead of called genotypes, and we have therefore included basic SNP discovery and genotype calling into ANGSD, using methods that efficiently can take advantage of estimated priors derived from GL based analyses. In ANGSD SNPs are inferred based on allele frequency estimation using a likelihood ratio test that can reject that the allele frequency is 0 [10]. We compare SNP calling using GATK (UnifiedGenotyper, default parameters), SAMtools (-q 10) and ANGSD based on 33 CEU samples from the 1000 genomes project [25]. ANGSD, SAMtools and GATK take into account the quality of the called bases (qscores) by modeling the uncertainty of possible genotypes, but differ in GL model, SNP calling criterion, filtering, etc (see [34]). In the commands below we perform SNP calling for all combinations of 1) p-value of site being variable 10^−6^,0.01**-snp_pval** 2) using local qscore recalibration with the BAQ model [35] -baq 3) SAMtools or GATK GL model **-GL**.



**Figure 4:**
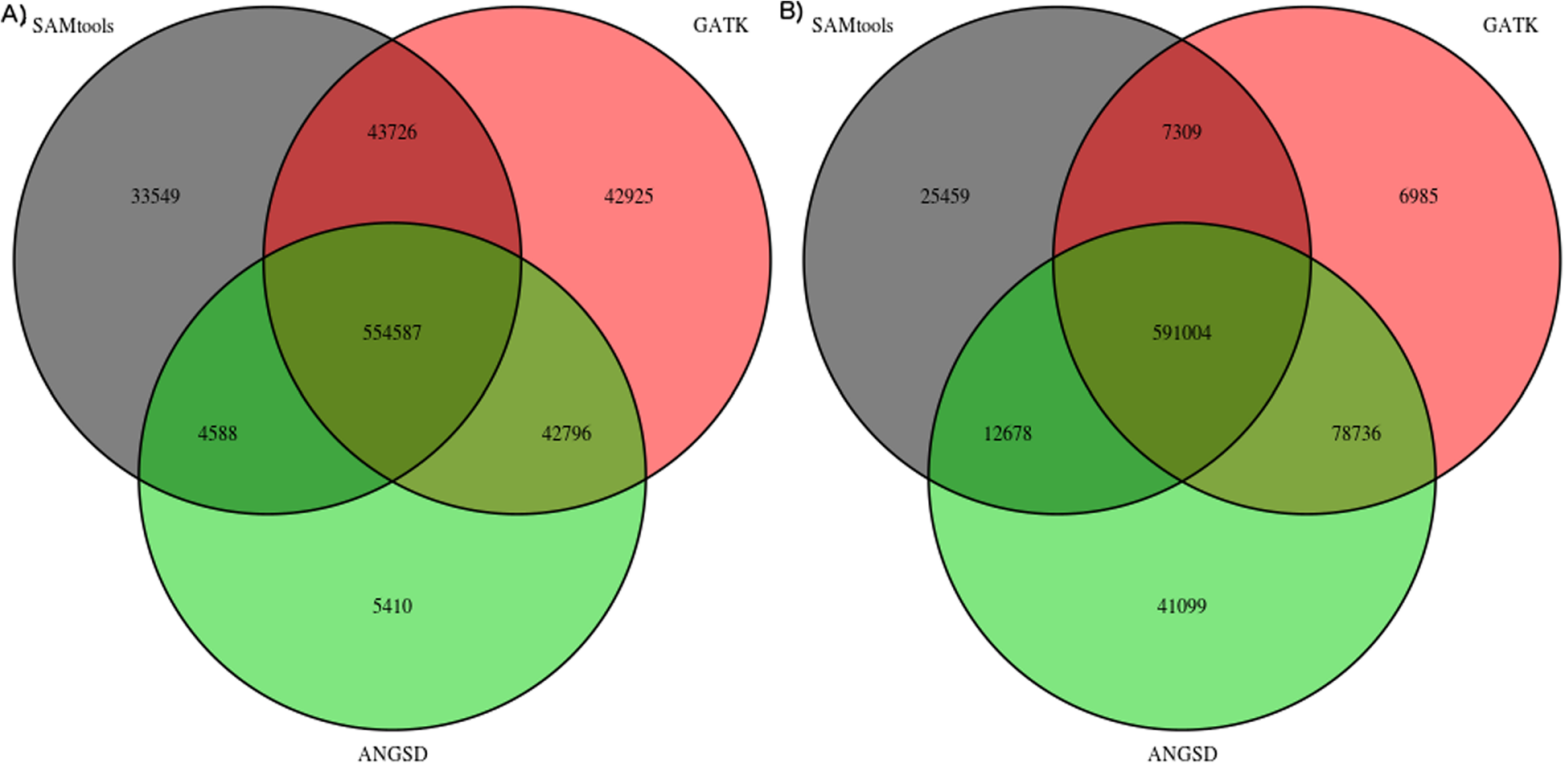
**Overlap between inferred SNPs with a critical p-value threshold of** ***10*** ^***−6***^ **and not using BAQ.** Venn diagram of the overlap between the SNP discovery for ANGSD, GATK and SAMtools for 33 CEU samples for chromosome 1. We used default parameters with GATK for SAMtools we discarded reads with a mapping quality below 10. For ANGSD we choose an p-value threshold of 10^−6^ and didn’t enable BAQ. In **A**, we used the SAMtools genotype likelihood model in ANGSD, in **B** we used the GATK model in ANGSD.

Venn diagrams of the overlap of sites are shown in Figure 4 (p-value <10^−6^, no BAQ), Additional file 4: Figure S4 (p-value <10^−2^, no BAQ), Additional file 5: Figure S5 (p-value <10^−2^, with BAQ), and Additional file 6: Figure S6 (p-value <10^−6^, with BAQ). Notice the difference in variable sites for the different GL models, and the decrease of variable sites when applying BAQ. When choosing a lenient p-value threshold (0.01) ANGSD infers more SNP sites than the other two methods when choosing a strict p-value threshold (10^−6^) fewer sites are called. In these analyses we removed sites that were called as homozygous for alternative alleles for SAMTools and GATK.

Assuming a segregating site is diallelic, there are 3 possible genotypes. In ANGSD we allow for 3 different methods for calculating genotype posteriors (GP), and we can define simple genotype calling criteria using these posteriors. We can either choose the genotype with the maximum posterior probability, or more sensibly, we can define a cutoff such that a genotype will be set to missing if it is below a certain threshold. Our 3 models for calculating GP are 1) assuming uniform prior (raw ML based on GL) (ML) 2) using an estimate of the population frequency as prior (AF) [10] 3) using the SFS as prior by calculating the genotype probabilities for an individual conditional on the information for all individuals [9] (SFS). We compared the three different ANGSD approaches with two existing tools for genotype calling (SAMtools,GATK) by using 31 CEU individuals that are part of the 1000genomes project and the HapMap project [36]. The exact commands used for this analysis in Additional file 7.

**Figure 5:**
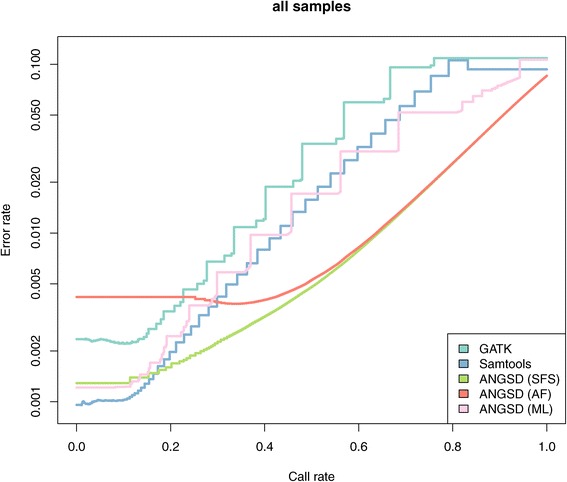
**Error rate vs call rate for called genotypes.** Error rate and call rates for genotype calls based on different methods. The error rate is defined as the discordance rate between HapMap genotype calls compared to the same individuals sequenced in the 1000 genomes. Genotype where called for all sites for all individuals for all methods. Each genotype call has a score which was used to determine the call rate. Due to the discrete nature of some of the genotype scores we obtain a jagged curve.

We include reference genome information for all methods even though ANGSD does not need the information. Additionally, we force all methods to call genotypes for all sites. Each genotype call is assigned a probability or quality score. A threshold can then be applied to remove low quality calls. For sites where the different method did not provide a genotype call we set the genotype as homozygous for the reference allele and give the call the worst possible quality score. The results for the 1,456,587 HapMap sites for all 5 methods are shown in Figure 5. The jaggedness of the SAMtools/GATK curves are due to the discretization of the phred scaled genotype qualities. We observe no big difference between the different methods for high call rates. For lower call rates we see that the ML method in ANGSD is somewhere between the GATK and SAMTools methods. For very low call rates we see that SAMtools outperforms the other methods.

### Computational speed

To compare the running time of ANGSD with existing tools we performed simple SNP discovery and allele frequency estimation for ANGSD, GATK and SAMtools. This we did with 50 samples, 100 samples and 200 samples (using the first 20 megabase region of human chromosome 21). For ANGSD and GATK we redid the analysis, but this time allowing for 2 and 4 cores (commands used are found in Additional file 7). The result is shown in Table 4. A fair comparison between tools is impossible for several reasons: 1) The tools perform slightly different analyses. 2) The storage subsystem might behave very different on different systems. 3) SAMtools is in its current version non thread-able in downstream analyses. 4) Difference in workload at runtime. In practice most analyses can be run in parallel over different chromosomes or even smaller regions, which makes the lack of threading in SAMtools more of a nuisance than a real problem. We would expect the runtime to be linear in the region size for all programs, and observed similar memory usage for all three tools. From the table we observe that ANGSD is faster in all scenarios, but we emphasize that can not conclude that ANGSD is generally faster, but we do not expect that it is significantly slower than SAMtools and GATK on any given system. We also note that the bottleneck for simple analyses is the file reading, and allocating an unlimited number of cores will not decrease the running time, but might actually increase it. This is what we observe for both ANGSD and GATK for this simple analysis. In a more complex analysis setting such as SAF estimation we would expect a better utilization of the threads. See Additional file 7 for commands used.

**Table 4:** **Computational speed of GATK,SAMtools and ANGSD**

	**SAMtools**	**GATK**	**ANGSD**	**GATK (2cores)**	**ANGSD (2cores)**	**GATK (4cores)**	**ANGSD (4cores)**
50 Samples	2722	1706	602	1744	1171	1765	1646
100 Samples	5097	4049	1270	4143	2457	4373	4013
200 Samples	10615	9672	2704	9951	5032	10330	7352

Wallclock time (not CPU) measured in seconds for different samples sizes and different number of allocated cores. Commands used are found in Additional file 7. We did the analysis twice (in different order) and picked the lowest value. Notice that the runtime for GATK and ANGSD does not decrease with 2 and 4 threads. This could be an indication that the file reading is the bottleneck.

## Conclusions

We have developed a fast program for analyses of NGS data that enable researchers to perform various analyses, particularly population genetic analyses that are not implemented in any other existing programs. For many of the analyses we use the full information of the data by avoiding genotype and SNP calling and instead basing analyses on GLs, calculated using different methods, typically using quality scores. This is especially useful for low-coverage data and for non-human organisms where imputation can not be performed reliably due to the lack of a reference population.

## Availability and requirements

- **Project name:** ANGSD (version 0.612 or higher)
- **Project home page:**http://www.popgen.dk/angsd, https://github.com/ANGSD.
- **Operating system(s):** Platform independent. But only tested on the Linux distribution Ubuntu.
- **Programming language:** c/c++.
- **Other requirements:** zlib. For some downstream analysis R is required.
- **License:** GPL version 2.
- **Any restrictions to use by non-academics:** None.

## Additional files

Additional file 1
**Figure S1.** True 2D site frequency spectrum. A heatmap of the two dimensional site frequency spectrum simulated on the basis of known genotypes using a demographic model to mimick 12 European individuals and 8 african samples. The estimated spectrum can be found in Additional file 2: Figure S2.

Additional file 2
**Figure S2.** Estimated 2D site frequency spectrum. A heatmap of the two dimensional site frequency spectrum estimated on the basis of genotype likelihoods for simulated genotypes. Data was simulated assuming a sequencing depth of 2X and an errorrate of 0.2%. The true estimates are seen in Additional file 1: Figure S1, and the difference between the true and the estimated can be found in Additional file 3: Figure S3.

Additional file 3
**Figure S3.** Difference between true proportions vs the estimated proportions. Plot of the estimated proportions and the true proportions. The estimated proportions are based on genotype likelihoods calculated assuming 2X sequencing depth and 0.2% error rate. The genotypes are simulated using msms and should reflect the difference European individuals (bottleneck followed by rapid expansion), and African individuals.

Additional file 4
**Figure S4.** Overlap between inferred SNPs, a critical p-value threshold of 10^−2^ and not using BAQ. Venn diagram of the overlap between the SNP discovery for ANGSD, GATK and SAMtools for 33 CEU samples for chromosome 1. We used default parameters with GATK for SAMtools we discarded reads with a mapping quality below 10. For ANGSD we choose an p-value threshold of 0.01 and didn’t enable BAQ. In A, we used the SAMtools genotype likelihood model in ANGSD, in B we used the original GATK GL model in ANGSD.

Additional file 5
**Figure S5.** Overlap between inferred SNPs, a critical p-value threshold of 10^−2^ with BAQ. Venn diagram of the overlap between the SNP discovery for ANGSD, GATK and SAMtools for 33 CEU samples for chromosome 1. We used default parameters with GATK for SAMtools we discarded reads with a mapping quality below 10. For ANGSD we choose a p-value threshold of 0.01 and enabled BAQ. In A, we used the SAMtools genotype likelihood model in ANGSD, in B we used the GATK model in ANGSD.

Additional file 6
**Figure S6.** Overlap between SNP sites, a critical value of 10^−6^ with BAQ. Venn diagram of the overlap between the SNP discovery for ANGSD, GATK and SAMtools for 33 CEU samples for chromosome 1. We used default parameters with GATK for SAMtools we discarded reads with a mapping quality below 10. For ANGSD we choose a p-value threshold of 10^−6^ and enabled BAQ. In A, we used the SAMtools genotype likelihood model in ANGSD, in B we used the GATK model in ANGSD.

Additional file 7
**Commands used for some of the analyses.** Text file containing the commands used in various analysis in the text. We used SAMtools version 0.1.19-44428cd, and GATK version 2.4-7-g5e89f01.
