# Supplementary figures and images for: ANGSD: Analysis of Next Generation Sequencing Data
Source: BMC Bioinformatics. 2014 Nov 25;15(1):356. doi: 10.1186/s12859-014-0356-4 (PMC4248462; doi:10.1186/s12859-014-0356-4)

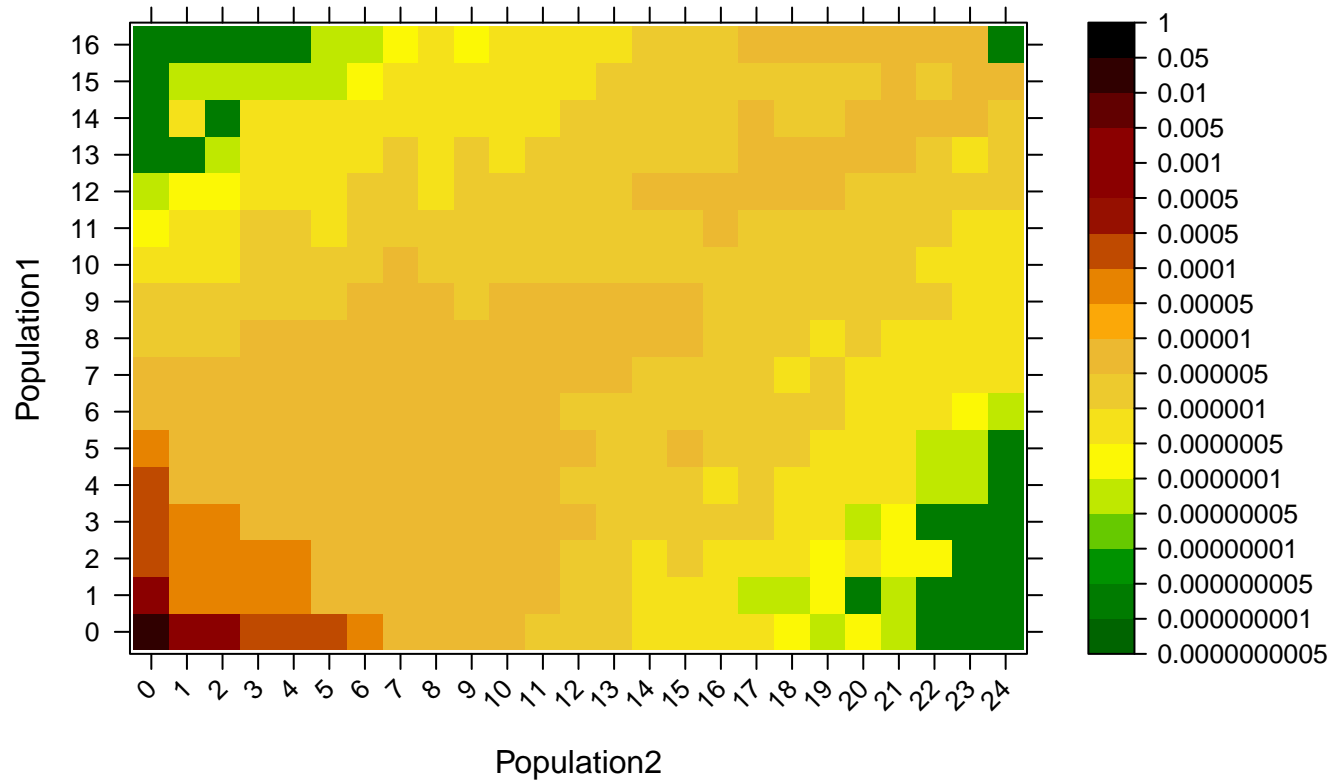

Supplement: Additional file 1 — Figure S1. True 2D site frequency spectrum. A heatmap of the two dimensional site frequency spectrum simulated on the basis of known genotypes using a demographic model to mimick 12 European individuals and 8 african samples. The estimated spectrum can be found in Additional file 2: Figure S2. [file 12859_2014_356_MOESM1_ESM.pdf]

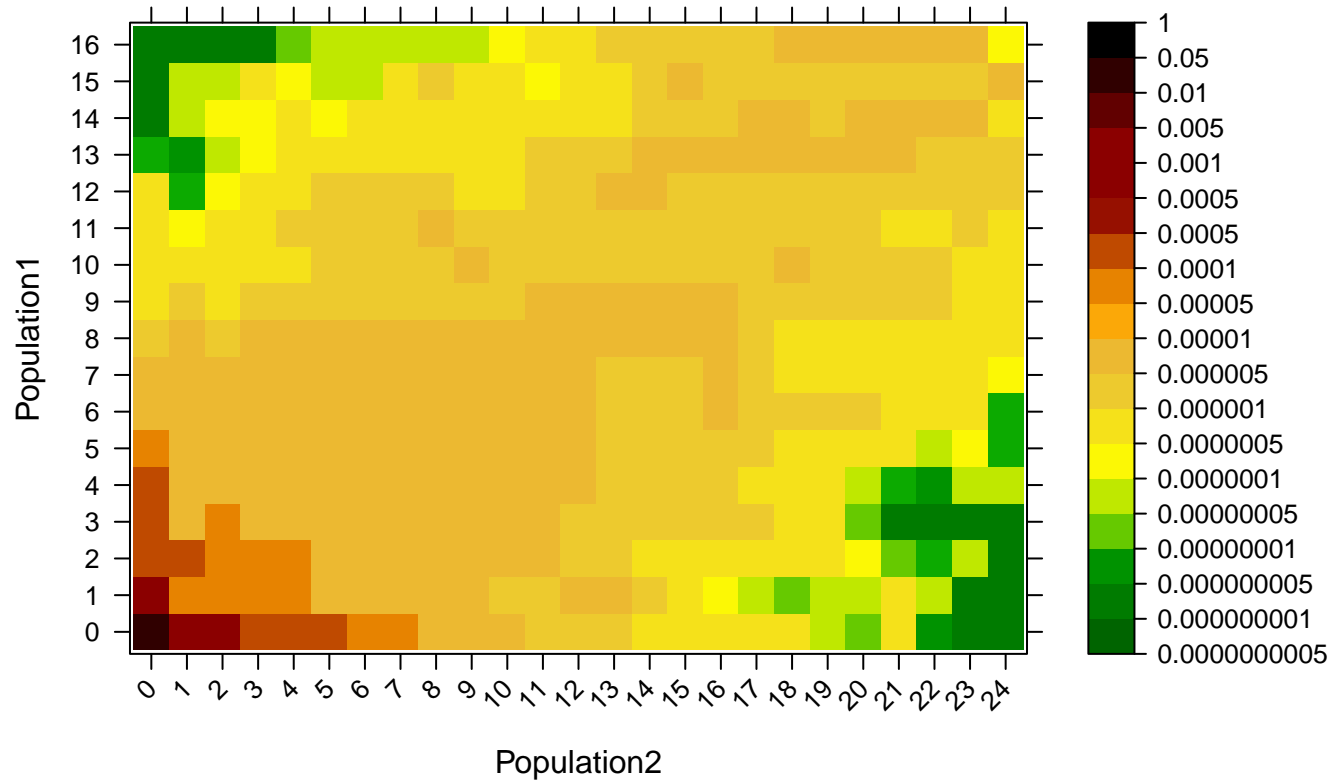

Supplement: Additional file 2 — Figure S2. Estimated 2D site frequency spectrum. A heatmap of the two dimensional site frequency spectrum estimated on the basis of genotype likelihoods for simulated genotypes. Data was simulated assuming a sequencing depth of 2X and an errorrate of 0.2%. The true estimates are seen in Additional file 1: Figure S1, and the difference between the true and the estimated can be found in Additional file 3: Figure S3. [file 12859_2014_356_MOESM2_ESM.pdf]

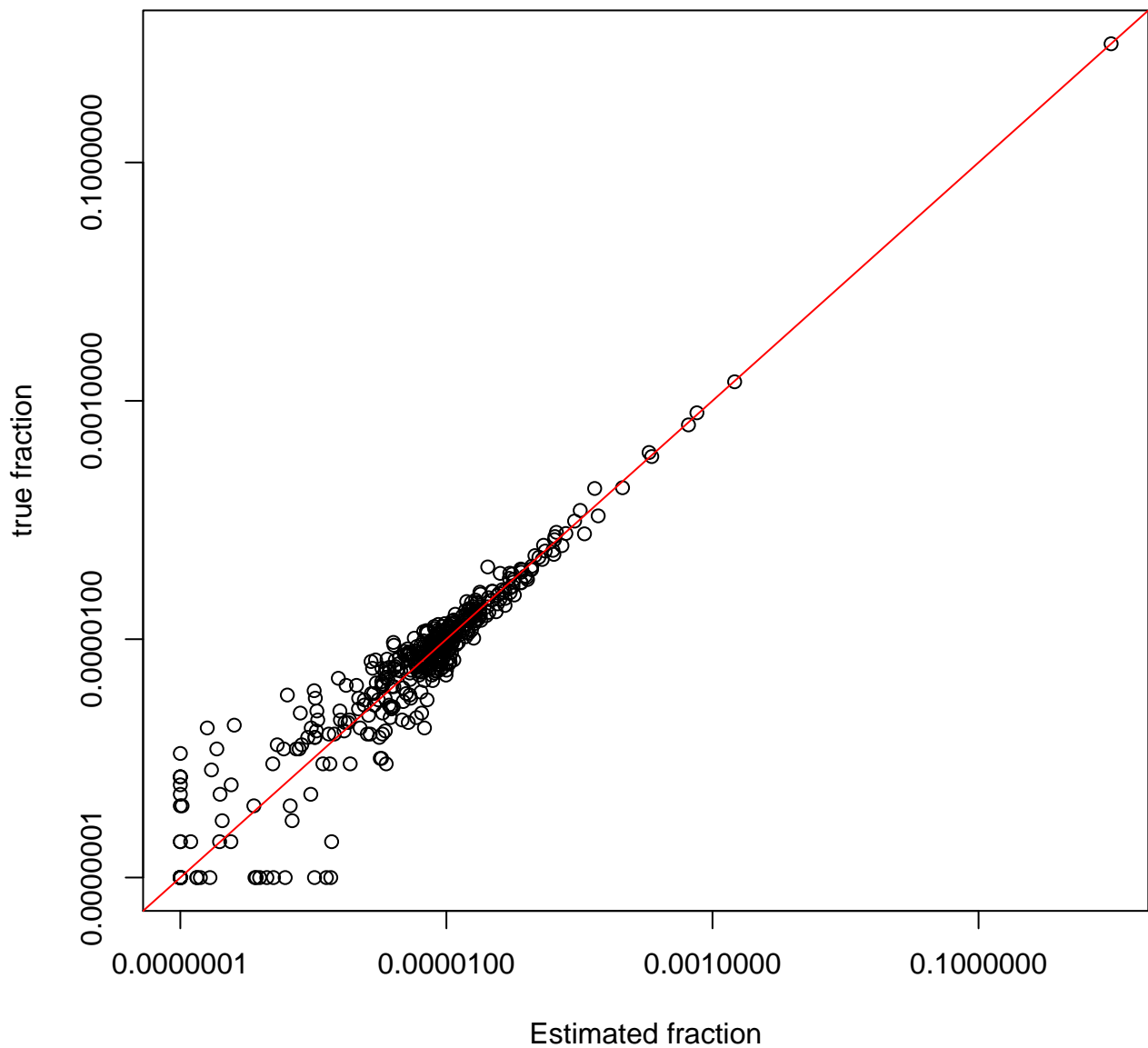

Supplement: Additional file 3 — Figure S3. Difference between true proportions vs the estimated proportions. Plot of the estimated proportions and the true proportions. The estimated proportions are based on genotype likelihoods calculated assuming 2X sequencing depth and 0.2% error rate. The genotypes are simulated using msms and should reflect the difference European individuals (bottleneck followed by rapid expansion), and African individuals. [file 12859_2014_356_MOESM3_ESM.pdf]

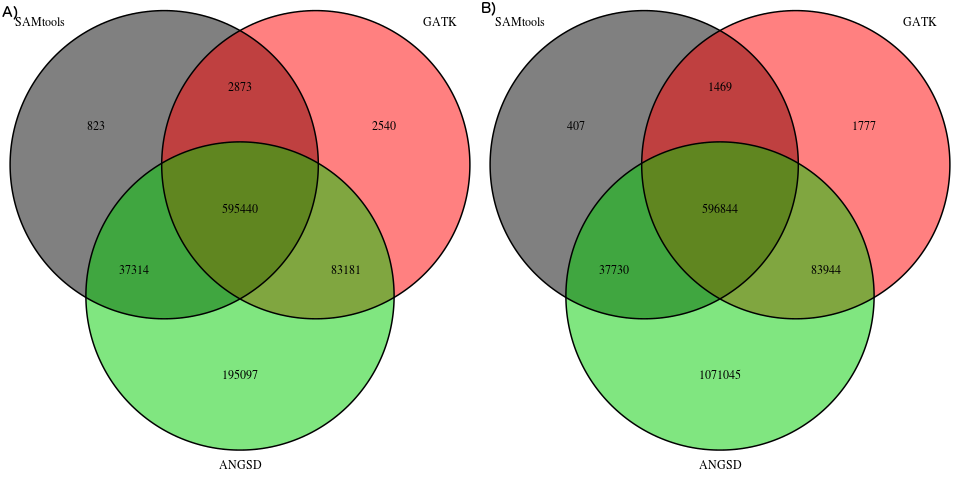

Supplement: Additional file 4 — Figure S4. Overlap between inferred SNPs, a critical p-value threshold of 10−2 and not using BAQ. Venn diagram of the overlap between the SNP discovery for ANGSD, GATK and SAMtools for 33 CEU samples for chromosome 1. We used default parameters with GATK for SAMtools we discarded reads with a mapping quality below 10. For ANGSD we choose an p-value threshold of 0.01 and didn’t enable BAQ. In A, we used the SAMtools genotype likelihood model in ANGSD, in B we used the original GATK GL model in ANGSD. [file 12859_2014_356_MOESM4_ESM.png]

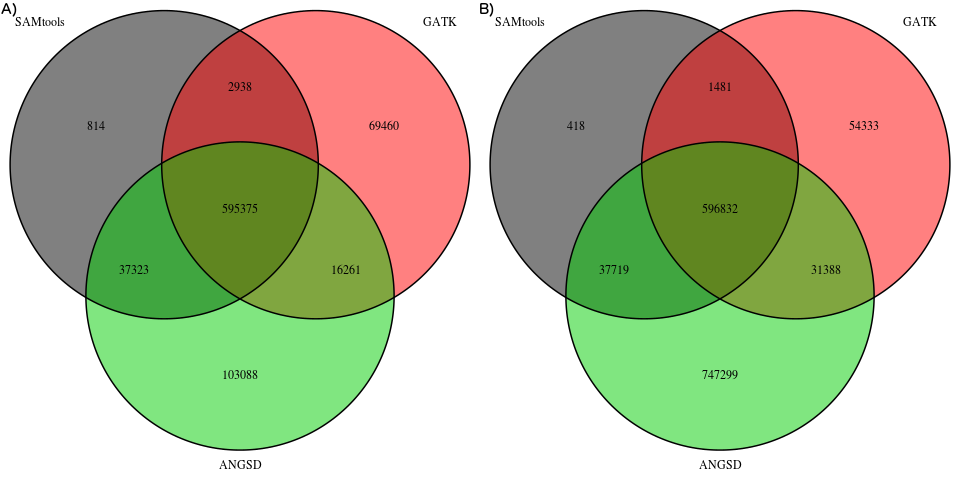

Supplement: Additional file 5 — Figure S5. Overlap between inferred SNPs, a critical p-value threshold of 10−2 with BAQ. Venn diagram of the overlap between the SNP discovery for ANGSD, GATK and SAMtools for 33 CEU samples for chromosome 1. We used default parameters with GATK for SAMtools we discarded reads with a mapping quality below 10. For ANGSD we choose a p-value threshold of 0.01 and enabled BAQ. In A, we used the SAMtools genotype likelihood model in ANGSD, in B we used the GATK model in ANGSD. [file 12859_2014_356_MOESM5_ESM.png]

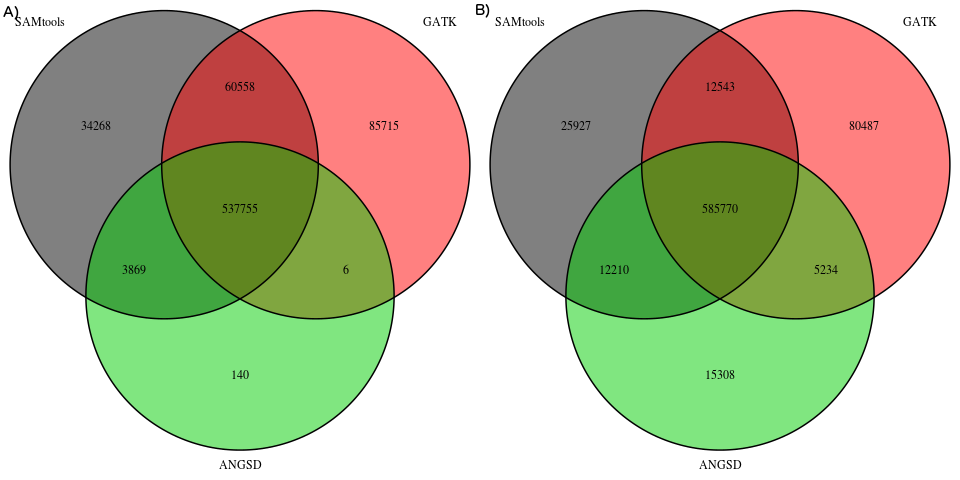

Supplement: Additional file 6 — Figure S6. Overlap between SNP sites, a critical value of 10−6 with BAQ. Venn diagram of the overlap between the SNP discovery for ANGSD, GATK and SAMtools for 33 CEU samples for chromosome 1. We used default parameters with GATK for SAMtools we discarded reads with a mapping quality below 10. For ANGSD we choose a p-value threshold of 10−6 and enabled BAQ. In A, we used the SAMtools genotype likelihood model in ANGSD, in B we used the GATK model in ANGSD. [file 12859_2014_356_MOESM6_ESM.png]
